# ASO Author Reflections: Diagnostic Accuracy of Nipple Aspirate Fluid Cytology in Asymptomatic Patients and Its Predictive Validity on Future Risk of Breast Cancer: A Meta-Analysis and Systematic Review of the Literature

**DOI:** 10.1245/s10434-020-09355-z

**Published:** 2020-11-18

**Authors:** Natasha Jiwa, Zoltan Takats, Daniel Richard Leff

**Affiliations:** 1 Department of Surgery and Cancer, Imperial College London, London, UK; 2 Department of Breast Surgery, Imperial Healthcare Trust, London, UK

## Past

Nipple aspirate fluid (NAF) is thought to reflect the microenvironment of a developing breast cancer and therefore is considered an important biofluid to interrogate for diagnostic purposes.^1^ Cytopathology as a technique was invented and used in the 1920s by George Papanicolaou for early diagnosis of reproductive tract cancer, whereby he was able to readily distinguish between normal and malignant cells in the cervix.^2^ His technique has been used since the 1950s as a “gold standard” diagnostic method for women presenting with symptomatic nipple discharge of the breast. Its potential use as a screening tool has been investigated in the past,^3^ but its diagnostic accuracy for use as a screening tool remains unquantified.

## Present

A meta-analysis of all English language research studies was performed, providing diagnostic information on the cytology of NAF across three databases and taking into account their quality scoring. The authors concluded that the diagnostic accuracy of NAF showed a pooled specificity of 0.97 (95% confidence interval [CI], 0.97–0.98) and a sensitivity of 0.64 (95% CI 0.62–0.66). Of the patients who produced a sample across all studies, 38.97% provided an inadequate sample for cytologic analysis.^4^ The key implication of this report is that NAF cytology is limited by poor sensitivity secondary to pauci-cellular material. This limitation therefore questions its role as a screening tool in clinical practice. The wider implications of this meta-analysis lie in what other methods of interrogating nipple aspirate fluid exist, either as stand-alone tests or indeed as adjuncts to current screening pathways for breast cancer.

## Future

In the search for superior methods of nipple fluid assessment, emerging technologies for fluid biomarker analysis must supersede the current diagnostic accuracy of NAF cytology while taking into account other important factors such cost effectiveness, reproducibility of results, user dependency, and turn-around time in the laboratory. Future work should focus on addressing these issues if cytology is to be replaced in clinical practice.
